# Hepatocellular Metabolic Profile: Understanding Post-Thawing Metabolic Shift in Primary Hepatocytes In Vitro

**DOI:** 10.3390/cells14110803

**Published:** 2025-05-29

**Authors:** Salvator Palmisano, Joshua D. Breidenbach, Brett R. Blackwell, Tara Harvey, Kes A. Luchini, M. Grace Thornhill, Erick S. LeBrun, Phillip Mach, Trevor Glaros, Emilio S. Rivera

**Affiliations:** 1 Biochemistry and Biotechnology Group (B-TEK), Bioscience Division, Los Alamos National Laboratory, Los Alamos, NM 87545, USA; spalmisano@lanl.gov (S.P.); joshuab@lanl.gov (J.D.B.); blackwell@lanl.gov (B.R.B.); tharvey@lanl.gov (T.H.); kluchini@lanl.gov (K.A.L.); thornhilmg@lanl.gov (M.G.T.); mach@lanl.gov (P.M.); 2 LMI, Colorado Springs, CO 80919, USA

**Keywords:** primary human hepatocytes (PHHs), liver metabolism, LC-MS/MS metabolomics, metabolic profiling, in vitro liver models, post-thawing hepatocyte stability

## Abstract

Primary human hepatocytes (PHHs) are widely used as in vitro models for liver function and drug metabolism studies, yet their metabolic stability post-thawing remains an open question. To better characterize early metabolic changes, we conducted a time-course experiment using liquid chromatography-tandem mass spectrometry (LC-MS/MS) to analyze metabolic shifts in PHHs cultured in suspension. Unexposed and exposed (acetaminophen-treated) samples were evaluated, and TITAN analysis was applied to determine the time point of maximal metabolic change at both individual metabolite and global metabolic profile levels. Our results indicate that the majority of metabolic shifts occur within the first five hours post-thawing. In the early culture time points, substantial metabolic overlap was observed between unexposed and exposed cells, suggesting a conserved biological response likely related to cellular recovery. However, at later time points, metabolite profiles diverged, with acetaminophen treatment-specific metabolic changes emerging, potentially reflecting differences in homeostatic restoration versus hepatotoxic responses. Our study highlights the importance of considering early post-thawing metabolic dynamics in experimental design and offers insights for optimizing hepatocyte culture protocols to better replicate in vivo physiological conditions.

## 1. Introduction

The liver plays a major role in numerous physiological processes, largely regulating the absorption and metabolism of nutrients and exogenous compounds. Due to its role in first-pass metabolism, the liver receives blood from the stomach, spleen, pancreas, small intestines, and colon before circulating back to the heart. It is positioned to participate not only in exocrine functions like bile production, but endocrine functions. These secreted products flow from the liver sinusoids via the central veins back to the heart for circulation throughout the body, participating in functions including the immune system, platelet production, blood pressure regulation, and appetite [1,2].

Because of the liver’s crucial roles in human physiology, benchtop models representative of human liver remain paramount to advancements in medicine and basic science. Hepatocytes are the major cell type of liver tissue, expressing the full repertoire of drug metabolizing enzymes and transporters, and are involved in all major liver functions [3,4]. Primary human hepatocytes do not expand reliably in culture, and therefore are expensive, and sources can be inadequate for large-scale studies. To circumvent these supply issues, stem cells can be expanded and differentiated, although these cells, termed hepatocyte-like, do not completely recapitulate the primary human hepatocyte, reducing the utility of this approach [5]. Thus, human primary hepatocytes are still often the model of choice for benchtop liver studies and are routinely used for the prediction of metabolic clearance; however, primary human hepatocytes still have shortcomings in their longevity and translatability [6]. The expression of major metabolic enzymes and transporters steadily falls after cell isolation and is significantly reduced as soon as 2 h [4]. Although hepatocytes cultured in suspension have demonstrated greater stability than those cultured in adherent conditions, variability is still observed [7].

Despite the shortcomings, primary hepatocytes remain the best choice for numerous assays such as drug metabolism, hepatotoxicity, and disease modeling assays [8,9,10]. Therefore, there is a need for in-depth characterization of primary human hepatocytes in the initial hours of culture, in which they are typically used for study [11]. Herein, we utilized mass spectrometry-based metabolomics approaches to investigate cellular changes within human primary hepatocytes cultured in suspension for 24 h, with and without a chemical exposure challenge to acetaminophen (APAP). APAP metabolic effects are well-characterized in liver cells in both therapeutic and hepatotoxic doses, with well-defined metabolic effects, enabling investigation of differences between normal metabolic changes in thawed hepatocytes, as compared to metabolic changes in challenged cells over time [12,13]. This exploratory study investigates changing metabolism in the early culture phase of primary hepatocyte cells, taking a global, untargeted approach to characterize metabolomic shifts and their change points—insights that can inform future study designs, including toxicology assessments in this cell type. This approach affords broad trends to be investigated without limiting the study to select metabolites that can be identified. We hypothesize that metabolic changes early on post-thawing may involve conserved metabolic shifts, due to the cellular recovery processes where the hepatocytes regain their viability and functional capabilities, independent of chemical exposure. However, later in the time course, we expect a divergent metabolic shift between treatments that we expect to be specific to biological differences based on exposures.

## 2. Materials and Methods

### 2.1. Culture of Human Hepatocytes

Mixed gender XenoTech human 10-Donor pooled cryosuspension primary hepatocytes (XenoTech; Kansas City, KS, USA; Cat#HPCH10, lot #2210104) were cultured according to the manufacturer’s protocols. Briefly, prior to thawing the cells, OptiThaw (XenoTech; Kansas City, KS, USA; Cat#K8000) and William’s E medium (Gibco; Waltham, MA, USA; Cat# A1217601) were warmed to 37 °C. Cells were thawed in a 37 °C water bath for 80 s and reconstituted in OptiThaw medium. The cell suspension was centrifuged for 5 min at 300× *g*, and the supernatant was removed. The cell pellet was reconstituted in 10 mL William’s E medium and counted via hemacytometer with trypan blue staining, which yielded a viability of >90%. Cells were diluted in William’s E media and distributed into 24-well plates (Corning; 3524; Corning, NY, USA) at 3 × 10^5^ cells per well. Seeded cells were acclimated in a 37 °C 5% CO_2_ incubator for 15 min before experiments.

To assess functional metabolic activity, and as a positive perturbagen control, cells were exposed to APAP (Sigma Aldrich; St. Louis, MO, USA; CASRN 103-90-2, purity ≥ 98%). Unexposed cells were cultured in William’s E media alone. Exposed cells were subjected to a final concentration of 50 mM APAP in 0.1% acetonitrile (ACN) (Fisher Scientific; Hampton, NH, USA; Cat#A9554) in William’s E media. Plates were briefly mixed via orbital shaker and then placed back in a 37 °C CO_2_ incubator for up to 1440 min, with one plate collected at each time point (10, 30, 60, 120, 240, 360, 480, 720, and 1440 min). After each respective incubation, plates were washed three times with PBS (Invitrogen; Waltham, MA, USA; Cat#10010-049), pelleting cells via centrifugation for 5 min at 300× *g*. After the final wash, PBS was removed, and pelleted cells were flash frozen for downstream analysis.

### 2.2. Preparation for LC-MS/MS Analysis

Following washing and freezing of samples, samples were prepared for analysis by liquid chromatography tandem mass spectrometry (LC-MS/MS), as described previously [14]. Briefly, hepatocyte samples were suspended in lysis buffer consisting of acetonitrile/methanol/acetone (8:1:1) and vortexed for 30 s. Samples were incubated at −20 °C for 30 min to better precipitate proteins, then centrifuged at 10,000× *g* for 10 min at 4 °C to remove proteins. Supernatant was transferred to a new tube and dried in a Speedvac at room temperature. Samples were reconstituted in 100 µL of 0.1% formic acid in water, vortexed for 30 s, then placed on an ice bath for 15 min. Final samples were centrifuged at 10,000× *g* for 10 min at 4 °C, and supernatant transferred to an HPLC vial with a glass insert for LC-MS/MS analysis. A pooled sample was created for QC purposes by combining an equal volume of each sample into a single vial, and samples were immediately analyzed by LC-MS/MS.

### 2.3. LC-MS/MS

Samples were injected with a volume of 10 µL onto a Vanquish Horizon UHPLC (Thermo Fisher Scientific, Waltham, MA, USA) coupled with an ultra-high resolution Exploris 480 mass spectrometer (Thermo Fisher Scientific, Waltham, MA, USA). Reversed-phase chromatography was performed using a Cortecs T3 C18 column (2.1 mm × 150 mm, 1.6 μm particle size, Waters Corporation, Milford, MA, USA) with a flow rate of 400 μL/min over a 15 min acquisition time in both positive and negative ionization modes for analytes that ionize in positive and negative polarity, respectively. Mobile phases for reversed phase were 0.1% formic acid in water (A) or ACN (B), beginning at 2% B, increasing to 100% B at 10 min, washing at 100% B for 2 min, then re-equilibrating at 2% B for 3 min, for a total 15 min run. Spectral data for unknowns were collected in MS1 only, where the scan range was *m*/*z* 70–700 with a resolution of 120,000. MS2 spectra were acquired at 40,000 resolution in data-dependent mode on pooled samples only for compound identification using spectral library matching.

### 2.4. Data Processing

Data from positive and negative polarity acquisitions were separately processed using Compound Discoverer software v3.3 (Thermo Fisher Scientific, Waltham, MA, USA). Peak rating threshold was set to 4, a signal-to-noise (S/N) ratio of ≥1.5, and compounds needed to be detected in at least 10/13 batch QC pooled samples with a relative standard deviation (RSD) ≤ 30%. Extraction blanks were used for background compound identification. Compound Discoverer results were used for all subsequent analyses.

### 2.5. Statistical Analysis

All statistical analysis was performed in R (version 4.3.3) [15]. Shapiro–Wilk and Kruskal–Wallis tests were performed to determine if metabolite abundance was normally distributed and to determine whether significant differences exist at the metabolite level over the time course, using the R package stats (version 4.3.3) [16,17]. Threshold Indicator Taxa Analysis (TITAN) was performed using the TITAN2 package (version 2.4.3) also in R [18]. Features not seen in ≥3 samples were removed, and TITAN was used to determine metabolic change points over the time course.

Features that met TITAN purity and reliability criteria were determined from the metabolite dataset [19], using the default setting of 0.95 for both. Purity is defined by the consistent assignment of a feature as either increasing or decreasing in abundance, while reliability is defined by consistent statistical determination of the metabolic change point over time. Z-scores were calculated for metabolites, representing how far each metabolite’s response deviates from the mean, in standard deviations, and these Z-scores were aggregated to generate the SumZ value, which provides a community-level measure of how the overall metabolic profile shifts over time. Individual metabolite change points were determined using the zenv.cp output of TITAN, which captures the change point in terms of Z-score, indicating the time point at which that metabolite undergoes a substantial shift. Metabolic change points were investigated over arbitrary time segments to discern how metabolic changes differ over time. For the purposes of condensing the data and looking at metabolic responses in the early culture phase as compared to later culture phases, we segmented the data and explored individual metabolites whose change point occurred between 0–2 h, 2–4 h, and 4+ h.

## 3. Results

**Figure 1:**
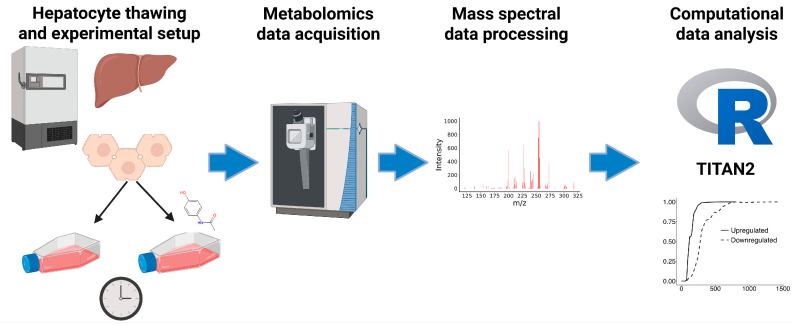
Workflow diagram highlighting experimental setup, mass spectrometry analysis, and computational analysis. The image was created Created in Biorender. Palmisano, S. (2025) https://BioRender.com/7cg0wfg (accessed on 26 March 2025).

Primary human hepatocytes were cultured for various amounts of time and analyzed by mass spectrometry-based metabolomics to elucidate the metabolic shifts over the typical culture period (Figure 1). In addition, APAP exposures were investigated over this time course to understand the functional change in metabolism of a known compound over time, as well as for comparisons between changes resulting from time alone, and “altered metabolism” due to a treatment effect. In this study, 1071 metabolic features were observed in the positive ionization mode, and 608 were observed in the negative ionization mode. All observed features were used for downstream statistical analyses.

### 3.1. Evidence of Hepatocyte Metabolic Restoration

**Figure 2:**
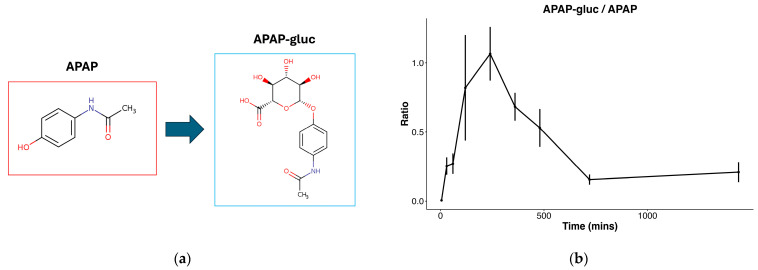
Figure describing APAP metabolism: (**a**) Common metabolic conjugation of APAP to glucuronide (APAP-gluc); (**b**) ratio of APAP-gluc to APAP throughout time course in hepatocytes exposed to APAP in 0.1% ACN.

To first confirm hepatocellular metabolic activity, we focused on identifying APAP and its metabolites in the exposed cells. The presence of APAP was identified at a level 2 of the metabolomics standards initiative via matching of fragmentation data to a metabolite MS/MS library, and acetaminophen glucuronide (APAP-gluc, a common hepatocellular conjugation product of APAP) at a level 3 via matching of precursor *m*/*z* to a metabolite database [20]. Both were confirmed to be present in only exposed samples. Manual confirmation of APAP-gluc was performed by determining that a prevalent in-source fragmentation of APAP-gluc was observed, producing the free APAP ion from the neutral loss of glucuronide, and further confirming the correct identification. The conjugation of APAP to APAP glucuronide is a well-established phase II metabolic pathway in the liver (Figure 2a), and the ratio of APAP glucuronide beginning at 0 (in APAP exposure treatment), sharply increases through 240 min, then decreases through 720 min (Figure 2b). The production of APAP glucuronide confirms cellular viability and metabolic activity, supporting the subsequent experimental findings.

### 3.2. Characterization of Metabolic Shift

A classical statistical approach to determine differences between groups (in our case, change between time points) is analysis of variance (ANOVA), which relies on the assumption of normality [21]. Metabolomics data are known to be noisy, and analysis of biological datasets often ignores assumptions of normality in statistical tests [22,23]. We performed the Shapiro–Wilk test to test for normality of each metabolite and observed that 1201/1665 (71.3%) have a *p*-value < 0.05, rejecting the assumption of normality and indicating that the majority of metabolites do not fit a normal distribution [16]. Therefore, we instead performed a Kruskal–Wallis test to determine whether there were significant differences between the distributions of samples from more than two groups, without assuming normality [17]. Results from Kruskal–Wallis indicated that 206/1675 (12.3%) features are determined significant (Figure S1), indicating that there is a change between time points at the individual metabolite level. 

While Kruskal–Wallis can indicate that a metabolite is changing across the time course, it will not determine where the time point of change is occurring. As our goal was to characterize changes in hepatocyte metabolism over time, including at what time point the maximum change was observed at a whole metabolome level, as well as for individual metabolites, we performed TITAN analysis as an alternative statistical approach. Designed to detect environmental thresholds (transition points), TITAN allows us to detect points of relatively rapid change as a response to an environmental variable. In our case, the environmental variable is time; thus, this analysis allows us to identify specific time points of greatest change at the individual metabolite and the entire metabolome levels. Since metabolomics data may not follow traditional distributional assumptions, bootstrapping allows for a non-parametric way to estimate metabolic change points.

**Figure 3:**
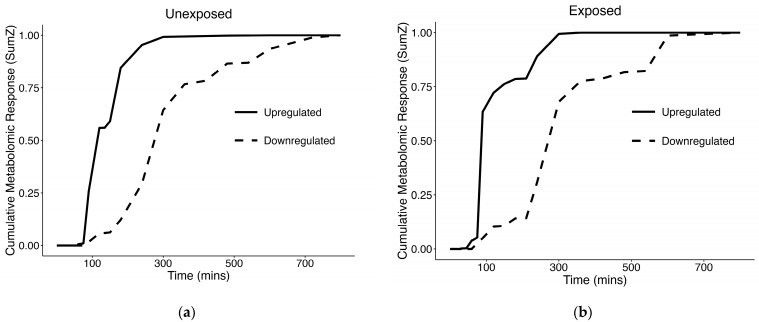
TITAN SumZ plots indicating changing metabolism over the time course, looking at the following: (**a**) Unexposed samples; (**b**) samples exposed to APAP in 0.1% ACN.

At the individual metabolite level, we observed 504 changing metabolites that passed purity and reliability thresholds in unexposed samples, and 227 in exposed samples (Table 1). In SumZ plots of the filtered metabolites which passed the purity and reliability thresholds for both unexposed and exposed cells (Figure 3), we observed a rapid shift in upregulated metabolites, between 0–2 h. The upregulated metabolites in both of these experimental conditions represent the majority of strongly responsive metabolites, with 79.0% (398/504) in unexposed cells and 86.3% (196/227) in exposed cells, respectively (Table 1). Named compounds in our study were identified at a level 3 of the metabolomics standard initiative via matching of precursor *m*/*z* to a metabolite database; therefore, structure names are indicated as tentative. Of the features assigned a molecular formula and determined by TITAN to be changing over time, the molecular formulas, tentative names, *m*/*z*, retention times, ion mode, time point of maximum change, and direction changing is shown in the supplement for unexposed (Table S1), and exposed samples (Table S2).

**Figure 4:**
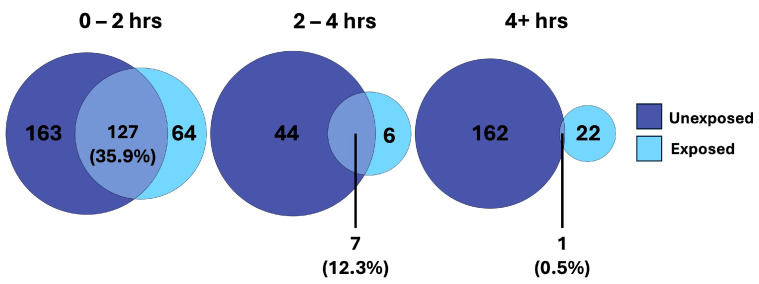
Venn diagrams demonstrating the overlap of TITAN filtered metabolites of unexposed and exposed hepatocytes, separated by time intervals across the time course experiment.

We observed considerable overlap in the features changing over time in both the unexposed and exposed cells, reflecting conserved changes unrelated to exposure (Figure 4). Considering time as a factor, we observed that the majority of the conserved changes occurred within the first two hours, with far less overlap further into the time course. This supports our hypothesis that conserved metabolic shifts are occurring early on, independent of toxic chemical exposure. However, in the later phase of the time course (2–4 h and >4 h), we observed a divergent metabolic shift between treatments that we expect are specific to the cellular response to APAP.

## 4. Discussion

Here we report the metabolomic profile of primary human hepatocytes during the initial hours of culture, which is the period of time in which these cells are commonly utilized for toxicology studies. To confirm functional metabolism in these cells, we identified an increasing APAP glucuronide to APAP ratio after exposure to APAP. Of note, the ratio of APAP-gluc to APAP increases steadily through the 120 min time point, followed by a slow decline through the remainder of the time course study. These results indicate that while the conjugation of APAP to APAP-glucuronide takes place early on, the observed decrease in later time points may be the result of further downstream metabolism after the 120 min time point, further suggesting that much of APAP-related metabolism takes place in the later phases of this study. It is important to note that since the primary focus of this study is on the unexposed condition, we do not distinguish between the individual contributions of APAP and ACN to the effects observed in the exposed cells.

In the unexposed condition, cellular stress responses are expected upon initial culture, due to the mechanical and biochemical stress of cryopreservation. For example, DMSO, a common component of freezing medium, has been observed to cause oxidative stress through the production of reactive oxygen species [24,25,26]. In addition, cellular damage has been observed from the freezing process and the interruption of normal metabolic function [27]. It is known that the cellular viability of hepatocytes post-thawing is decreased as compared to cells from a fresh culture [28]. The temporal component of cell death post-thawing is largely unstudied, motivating this study. However, other hepatocyte studies have implicated a host of initiation sites of cellular death, including the cell membrane, nucleus, and mitochondria [29].

When looking at the SumZ plot in the unexposed cells (Figure 3a), our analysis suggests the majority of the maximal metabolic shift, which occurs merely from time alone, has occurred by the 4 h time point, particularly when looking at the upregulated metabolites, which represent the majority of changing metabolites. Potential metabolic processes that may become activated shortly after culture initiation include membrane repair and reorganization, mitochondrial recovery and energy restoration, activation of stress response pathways, and resumption of metabolic activities. We hypothesized that these metabolic processes would be conserved in the early culture phase, while diverging over time due to treatment-specific effects. Taking a deeper look into the changed metabolites shared between unexposed and exposed cells, we observed substantial overlap early on. Interestingly, this overlap decreased over time (Figure 4). This suggests a conservation of metabolism early on, which may be associated with the biological processes involved in cellular recovery. Later in the time course, we observe less commonality in the changing metabolites between unexposed and exposed cells, indicating that the recovery process has largely stabilized, and the changing metabolites have become more treatment-specific.

Unintuitively, greater than twice the number of changing metabolites was observed in the unexposed cells compared with the exposed cells. A possible explanation may be that the hepatotoxic effects of APAP resulted in a dampened response to the otherwise typical recovery from cryopreservation, impairing aspects of metabolic recovery and restoration of cellular homeostasis.

Interestingly, it has been reported that APAP overdose toxicity occurs in two phases: the initial phase (0–2 h) involving activation of liver phase II biotransformation enzymes, and the second phase (3–5 h), which is dependent on the mitochondrial permeability transition, as well as release of alanine transaminase (ALT) [30]. Our results also suggest there may be a “biphasic” change in metabolism. This can be observed in the SumZ plot of exposed cells (Figure 3b) which suggests there may be multiple phases of metabolic transition, as indicated by rapid change in metablome composition in the early culture phase (0–2 h), followed by a slight plateau before another later response in metabolic shift.

While tentative, some structural annotations that changed over time illustrate potential links to known liver metabolism. For example, features matching citric acid and succinic acid relate to the TCA cycle, and other metabolites tentatively identified as L-valine and L-methionine point to branch-chained amino acid and sulfur metabolism, respectively [31,32,33]. These illustrative associations may help generate future hypotheses, and as untargeted metabolomics advances and structural annotation improves, this dataset can be revisited to gain deeper insight into which metabolic pathways shift over time.

One limitation of our study is that it does not isolate the impact of cell harvest (e.g., collagenase treatments, harvest time) from the freeze-thaw portion of preparation. While this would be an insightful comparison, as most researchers do not have access to these types of samples, many researchers will use commercially sourced frozen primary human hepatocytes as the gold standard for in vitro culture systems that recapitulate in vivo human liver characteristics [34]. Therefore, this study may not fully reflect the biology of freshly harvested, unfrozen cells. It may also have limited applicability to clinical contexts such as liver transplantation, where machine perfusion offers a more physiologically relevant environment for maintaining cells ex vivo [35]. Another limitation of this study is that the primary human hepatocytes, by definition, do not include all cell types of the human liver, which contribute to the differences between the in vitro model and the physiological environment.

Additional avenues for exploration include further investigation of different metabolic processes to better characterize metabolic response post-thawing. For example, performing proteomics analysis over a similar time course, which may demonstrate changes in antioxidant enzymes associated with hepatocellular function, such as glutathione and cytochrome P450 enzymes, or targeted investigation of biomarkers of apoptosis in hepatocyte cultures may provide valuable insights into the metabolic changes taking place. Also, increasing the confidence level of identification of metabolites from untargeted metabolomics experiments remains a challenge, and future progress in this area would support improved biological interpretation and characterization of metabolic shifts. Additional evidence gained from such studies may further inform and separate processes of cellular recovery, cellular response to toxicity, and potential loss of cellular viability, and have the potential to more accurately inform hepatocyte thawing protocols for in vitro experiments.

## 5. Conclusions

We present a metabolomics dataset of the observed shifts in the early culture phase of primary human hepatocytes, presumably due to the processes involved in cellular recovery from cryopreservation and the loss of function due to inadequate replication of the native environment. Therefore, this work provides some insight into informing optimal protocols for in vitro hepatocyte experiments. Specifically, the results of this study indicate that a high level of metabolic change is occurring early on in culture, and that treatment effects like those of acetaminophen exposure may take place after the first 120 min of cell culture. These results suggest that a longer acclimatization period of 2 h may provide clearer insight into insult-related changes compared with early culture phase adaptation. Importantly, the metabolomics dataset published with this manuscript will continue to inform on the shifts exhibited by hepatocytes as more confident metabolomics data processing and identifications are made possible. While hepatocyte models are undoubtedly important for toxicological studies, there remain many challenges to their use, and this work may serve as a critical resource moving forward.

## Figures and Tables

**Table 1:** Results of TITAN demonstrating the features that passed the purity and reliability thresholds as changing metabolites, and then number of upregulated and downregulated metabolites.

Treatment	# Input Features	# Filtered Features	# Upregulated	# Downregulated
Unexposed	1675	504	398	106
Exposed	1679	227	196	31

## Data Availability

The original data presented in the study are openly available in Mass Spectrometry Interactive Virtual Environment (MassIVE) at accession number MSV000097484.

## Supplementary Materials

The following supporting information can be downloaded at: https://www.mdpi.com/article/10.3390/cells14110803/s1. Figure S1: Results from Kruskal-Wallis tests indicating the number of metabolites that demonstrated a significant shift during the unexposed samples time course; Table S1: Table reporting metadata for all features assigned a molecular formula and determined by TITAN to be changing over time in unexposed samples; Table S2: Table reporting metadata for all features assigned a molecular formula and determined by TITAN to be changing over time in exposed samples.
