# Supplementary material for: Hepatocellular Metabolic Profile: Understanding Post-Thawing Metabolic Shift in Primary Hepatocytes In Vitro
Source: Cells. 2025 May 29;14(11):803. doi: 10.3390/cells14110803 (PMC12154394; doi:10.3390/cells14110803)
Supplement: Supplementary file 1 [file cells-14-00803-s001.zip › Figure S1.pdf]

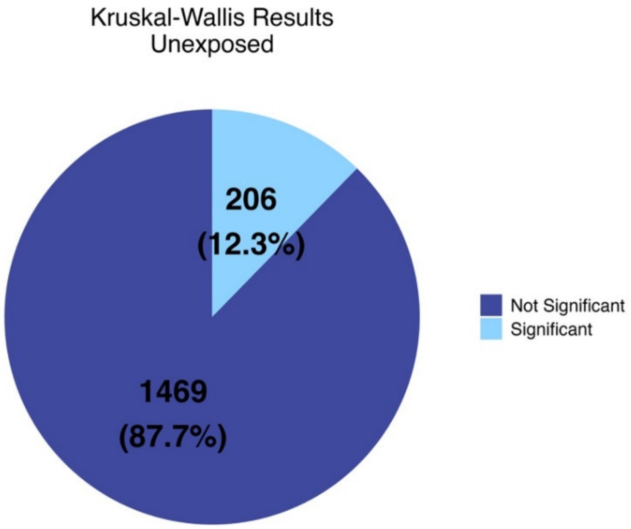

Figure S1: Results from Kruskal-Wallis tests indicating the number of metabolites that demonstrated a significant shift during the unexposed samples time course.
